# Performance of PRAME Immunohistochemistry in Distinguishing Classic Hodgkin Lymphoma from Anaplastic Large Cell Lymphoma and T-Cell Lymphoma

**DOI:** 10.3390/cancers18172875

**Published:** 2026-09-05

**Authors:** Xin Duan, Katherine N. Williams, Anne L. Chen

**Affiliations:** 1 Department of Pathology and Immunology, Washington University School of Medicine, 660 South Euclid Ave, Campus Box 8118, St. Louis, MO 63110, USA; 2 Barnes-Jewish Hospital, St. Louis, MO 63110, USA

**Keywords:** PRAME, Hodgkin, lymphoma, anaplastic

## Abstract

Background/Objectives: The differential diagnosis of classic Hodgkin lymphoma (CHL) versus anaplastic large cell lymphoma (ALCL) and other T-cell lymphomas (TCL) with Hodgkin-like features remains challenging in some cases, and may require TCR molecular testing with prolonged turnaround time. The limited literature has previously described PRAME (Preferentially Expressed Antigen in Melanoma) overexpression in Hodgkin cells and its absence in cutaneous T-cell lymphomas. The aim of this study was to examine the staining pattern of PRAME in CHL compared to the pattern in non-cutaneous ALCL and TCL. Methods: Twenty-four cases of CHL, 21 cases of ALK-negative ALCL, nine cases of ALK-positive ALCL and 11 cases of other TCL were stained for PRAME. Results: PRAME showed strong and diffuse (3+) expression in 80% of CHL cases while, in contrast, 0% of ALK-negative ALCL showed 3+ staining and 11% of ALK-positive ALCL showed 3+ staining. Non-ALCL TCL showed only 0 or 1+ staining. Conclusions: PRAME may be another helpful stain in the workup of CHL and its morphologic mimics.

## Simple Summary

Classification of lymphomas is important for appropriate treatment and clinical management decisions. One of the most difficult areas of hematopathology diagnosis is in the area of Hodgkinoid differentials. Existing stain panels are not entirely specific or definitive, with diagnoses sometimes depending on molecular testing, which has a longer turnaround time than stains. This study examines the performance of one additional stain option, in the form of PRAME, for this category of differentials.

## 1. Background

The diagnostic distinction between classic Hodgkin lymphoma (CHL) and its T-cell mimics remains one of the challenges in hematopathology, often requiring a comprehensive integration of morphology, immunophenotype, and molecular studies. Anaplastic large cell lymphoma (ALCL), particularly the ALK-negative variant, can present a significant diagnostic pitfall due to its expression of CD30 and the presence of large, pleomorphic “hallmark cells” that may resemble the Reed–Sternberg (RS) cells characteristic of CHL [1,2,3]. This diagnostic overlap is further complicated by rare cases of ALCL that demonstrate aberrant PAX5 expression, a marker traditionally utilized to confirm B-cell lineage in CHL and thereby stripping the pathologist of a reliable lineage-defining tool [1,4,5,6]. The frequent loss of standard T-cell antigens in ALK-negative ALCL can create a “null-cell” phenotype that is virtually indistinguishable from CHL on routine immunohistochemical panels [6]. Finally, expression of T-cell markers by CHL can further complicate evaluation [7].

Beyond ALCL, other T-cell neoplasms can mimic CHL. Nodal T-follicular helper (TFH) cell lymphomas, most notably angioimmunoblastic T-cell lymphoma (AITL), often harbor EBV-positive B-blasts that perfectly recapitulate the morphology and immunophenotype of RS cells within a polymorphous inflammatory background [8]. Emerging data also highlight peripheral T-cell lymphoma, not otherwise specified (PTCL-NOS) and certain cytotoxic T-cell lymphomas as entities that can adopt a Hodgkin-like growth pattern, often necessitating the use of extended panels including GATA3, TBX21, and TCR-gamma/delta stains [9]. Because these morphological and immunophenotypic overlaps are so extensive, definitive diagnosis frequently hinges on the detection of clonal T-cell receptor (TCR) gene rearrangements. However, the inherent technical limitations and long turnaround times (TAT) associated with molecular clonality testing can significantly delay the initiation of appropriate therapy, emphasizing the urgent need for more rapid, highly specific proteomic or genomic biomarkers to differentiate these biologically divergent but morphologically convergent malignancies.

PRAME (Preferentially Expressed Antigen in Melanoma) is a cancer–testis antigen (CTA) that functions as a repressor of retinoic acid receptor (RAR) signaling, promoting tumor cell survival and inhibiting differentiation [10,11]. While traditionally associated with melanoma, PRAME is an emerging diagnostic marker in hematologic malignancies. It is overexpressed in the HRS cells of CHL [12] and in subsets of diffuse large B-cell lymphoma (DLBCL) [13,14]. Recent data suggest PRAME expression may predict responses to immune checkpoint inhibitors and rituximab priming in DLBCL [15], and it has been correlated with treatment outcomes and specific microenvironmental features in CHL [16]. Conversely, PRAME is consistently absent in cutaneous T-cell lymphomas (CTCL) [17].

However, the expression profile of PRAME in systemic T-cell lymphomas, particularly ALCL, has not yet been systematically investigated. Such data could establish PRAME as a valuable surrogate marker for distinguishing CHL from its T-cell mimics in routine practice. This study evaluates the diagnostic utility of PRAME immunohistochemistry in differentiating CHL from non-cutaneous ALCL and other systemic T-cell lymphomas.

## 2. Methods

The study was conducted in accordance with the Declaration of Helsinki, and approved by the Institutional Review Board of Washington University in St. Louis (protocol code: 202502152 and date of approval: 14 March 2025).

### 2.1. Samples

The diagnostic criteria were based on the fifth edition of the World Health Organization (WHO) Classification of Haematolymphoid Tumours and International Consensus Classification (ICC). Formalin-fixed paraffin-embedded (FFPE) tissue blocks from patients diagnosed with classic Hodgkin lymphoma and T-cell lymphomas were identified retrospectively and consecutively from January 2019 to September 2025. All original case diagnoses were confirmed using current diagnostic criteria without any exclusions. For ALCL and other TCL, due to a limited number of cases identified in our archives, both core biopsies and excisional biopsies were included. For CHL, only excisional biopsies were included to adequately visualize enough HRS cells for PRAME staining. Treated and recurrent cases were included. Only non-cutaneous lymphomas were included except for one case in the TCL category for which the differential included possible mycosis fungoides involvement of the lymph node biopsy.

### 2.2. Histological and Immunohistochemical Study

**Figure 1:**
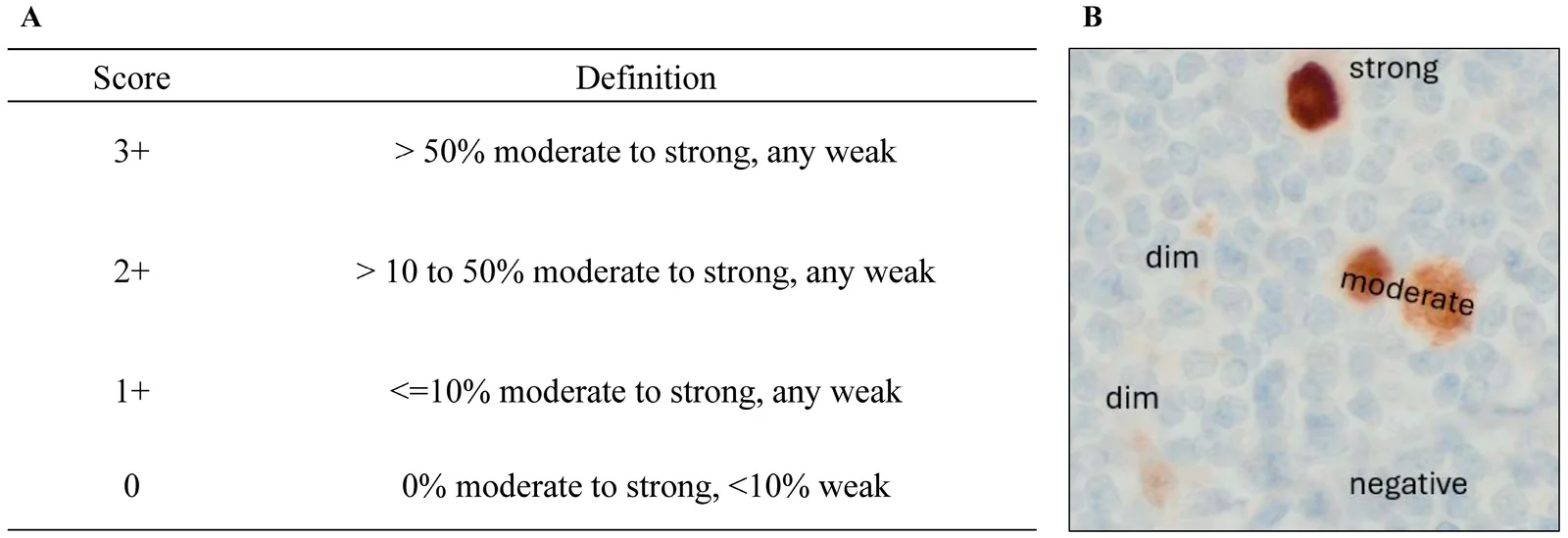
Semi-quantitative scoring system and representative image of PRAME expression. (**A**) Definition of semi-quantitative scores. (**B**) Representative image of different scores.

The original archived H&E (hematoxylin and eosin) slides from the diagnostic workup were used for analysis. Immunohistochemical staining for PRAME was performed as part of this study using Biocare clone anti-PRAME (EPR20330) according to the manufacturer’s protocol for Ventana Benchmark ULTRA (CC1 for 64 min, Pre-Primary Peroxidase Inhibitor, OptiView DAB IHC, pre-dilute)(Ventana Medical Systems, Inc., Tucson, AZ, USA). Two pathologists, blind to the diagnoses, independently scored each case. Since the neoplastic cell population would vary according to the diagnosis, with neoplastic cells in classic Hodgkin lymphoma being only large atypical/Hodgkinoid cells identified on H&E, each case was scored using two methods by the two blinded pathologists. Method 1 recorded the eyeballed percentage of all lesional cells that appeared positive for any intensity, and out of those positive cells, the percentage staining strongly, moderately or weakly. Method 2 recorded the eyeballed percentage of only large atypical/Hodgkinoid cells that appeared positive with any intensity, and out of those positive cells, the percentage that stained strongly, moderately or weakly (i.e., evaluating the case as if it were classic Hodgkin lymphoma). If no significant population of large atypical/Hodgkinoid cells was seen on H&E, only Method 1 scores were used. A third pathologist, who did not score the cases as above, and who was privy to the diagnoses, calculated the final defined scores in Figure 1A, using only Method 1 values if the diagnosis was CHL and using only Method 2 values if the diagnosis was ALCL or other TCL. For example, if a given case was CHL, and by Method 2, a blinded pathologist recorded the percentage of large atypical/Hodgkinoid cells having any staining as 52%, and out of those positive cells, the percentage with strong, moderate and weak staining as 50%, 50% and 0%, the final defined score from Figure 1 would be 3+, because 100% (50% +50%) of any-positive cells, comprising 52% of the atypical/Hodgkinoid cells, is “>50% moderate to strong, any weak” per Figure 1. The definition of negative, weak/dim, moderate and strong-intensity staining is shown in Figure 1B. The average of the final defined scores between the two blinded pathologists was used for the statistical calculations.

### 2.3. Statistical Analysis

Statistical analyses were performed using GraphPad Prism (v11.1.0, GraphPad Software, Boston, MA, USA). Categorical variables, including dichotomized PRAME immunohistochemical staining scores across different thresholds and the diagnosis of classic Hodgkin lymphoma (CHL) versus non-CHL T-cell lymphomas (TCL), were cross-tabulated into a 2 by 2 contingency table. Diagnostic performance metrics, including sensitivity, specificity, positive predictive value (PPV), and negative predictive value (NPV), were calculated to assess the operational utility of the PRAME stain. Fisher’s exact test (due the presence of low expected cell counts < 5) was utilized to evaluate the association between the diagnostic test (PRAME stain score) results and actual disease status. Continuous variables of the mean PRAME immunostaining scores among CHL, ALCL and other TCL were compared using the Kruskal–Wallis test followed by Dunn’s multiple comparisons test. For all analyses, a two-tailed *p*-value < 0.05 was considered statistically significant.

## 3. Results

### 3.1. Case Composition

A total of 65 cases were analyzed, including 24 CHL, 21 ALK-negative ALCL, 9 ALK-positive ALCL, and 11 other TCLs (comprising six peripheral/mature T-cell lymphomas not otherwise specified, three angioimmunoblastic T-cell lymphomas, and one TCL with T-follicular helper features). One of the six peripheral/mature TCLs not otherwise specified included a possible differential of lymph node involvement by mycosis fungoides. All CHL cases were excisional biopsies and comprised 13 nodular sclerosis, one mixed cellularity, one syncitial variant/nodular sclerosis, and nine with no specified subtype. The ALK-negative ALCL comprised 14 core biopsies, eight excisional biopsies and one bone marrow. The ALK-positive ALCL comprised two core biopsies and seven excisional biopsies. The other TCL category comprised four excisional biopsies, one bone marrow and six core biopsies. In the CHL cohort, two of the cases had positive immunoglobulin rearrangement (IGH) molecular test results. In the ALK-negative ALCL cohort, three cases had positive T-cell rearrangement (TCR) molecular testing results. In the ALK-positive ALCL cohort, one case had a polyclonal TCR molecular test result. In the other TCL cohort, three cases had positive TCR molecular test results and one had a polyclonal TCR molecular test result. For the remaining cases, no molecular test results were available. Table 1 provides the demographic characteristics of the cohorts.

### 3.2. PRAME Expression Patterns in Classic Hodgkin Lymphoma, Anaplastic Large Cell Lymphoma, and Other T-Cell Lymphomas

**Figure 2:**
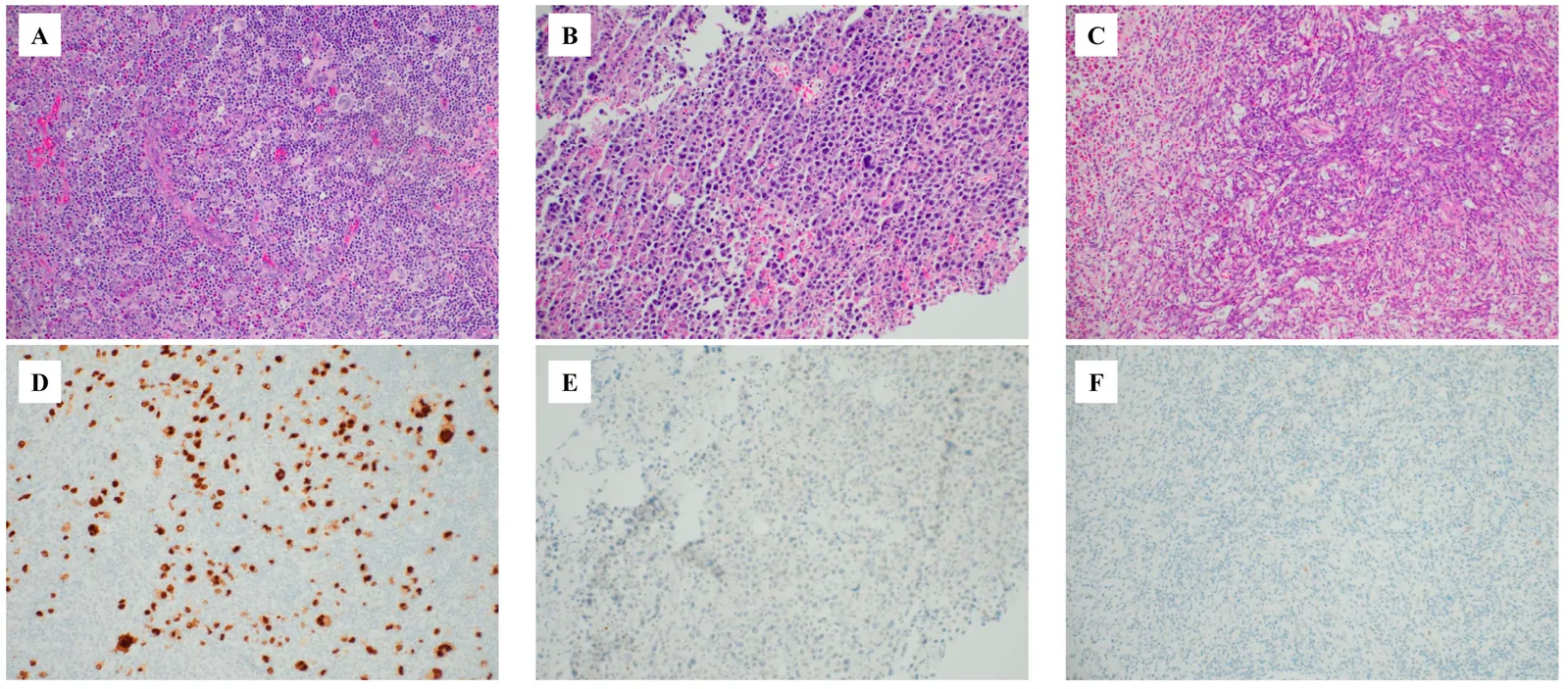
Representative cases of PRAME expression in CHL, ALCL, and other TCLs. (**A**–**C**) Representative images of H&E staining in CHL, ALCL, and other TCLs, respectively. (**D**–**F**) Representative images of PRAME immunohistochemistry in CHL, ALCL, and other TCLs, respectively; CHL shows strong and diffuse PRAME staining, whereas ALCL and other TCLs are negative. All images are at 200× magnification.

**Figure 3:**
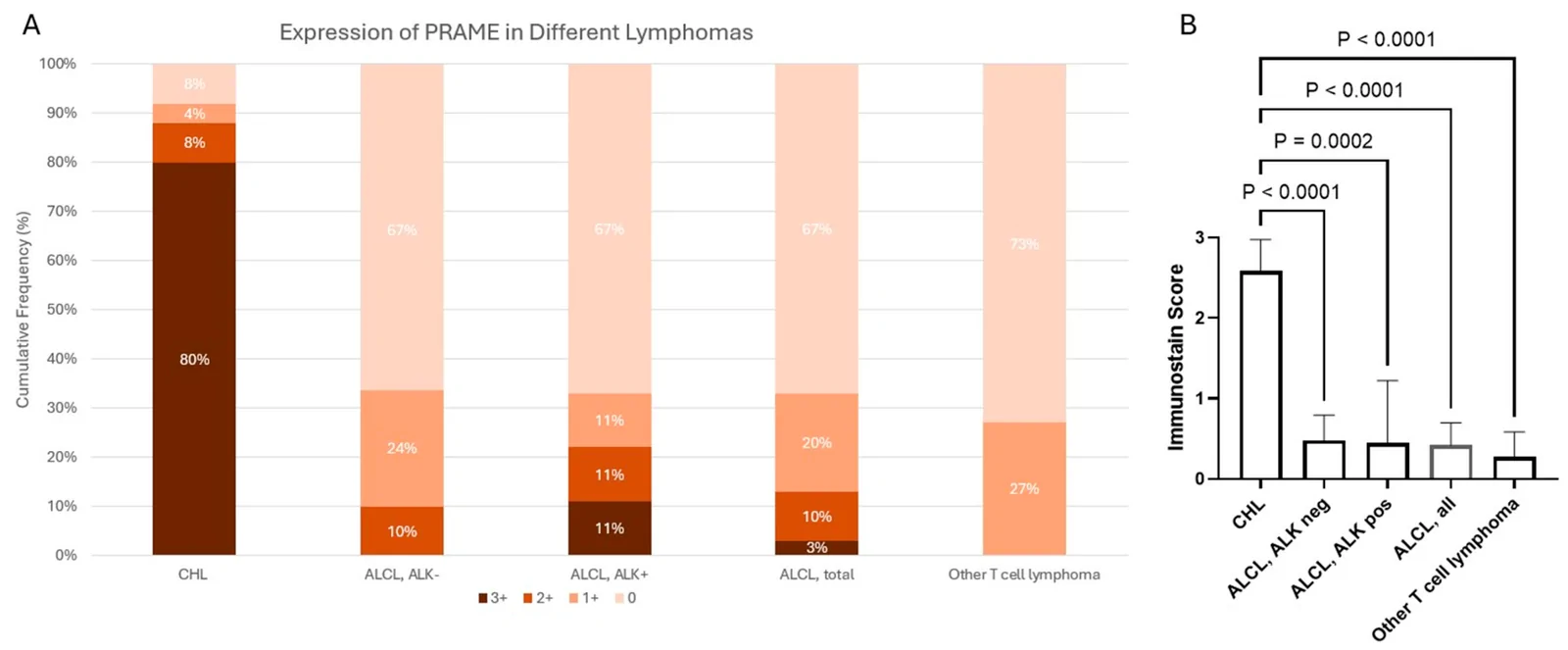
Characterization of PRAME expression levels and immunostaining scores in CHL, ALCL, and other TCL. (**A**) Expression of PRAME in different lymphomas: 80% of CHL show 3+ expression, whereas 0% of ALK-negative ALCL show 3+ expression. All of the non-ALCL TCL in this study showed only 0 to 1+ expression. (**B**) The mean immunostaining score for CHL is significantly higher than those for ALCL and other TCLs.

Distinct PRAME expression patterns were observed across the different lymphoma diagnostic categories. Classic Hodgkin lymphoma (CHL) cases exhibited strong and diffuse nuclear PRAME staining (Figure 2A,D), with a 3+ score observed in 80% of cases (Figure 3). In contrast, neoplastic cells in most mimic cases showed minimal to no expression: 0% of ALK-negative ALCL showed 3+ staining and 11% of ALK-positive ALCL showed 3+ staining. Similarly, other T-cell lymphomas (TCLs), including angioimmunoblastic T-cell lymphoma (AITL), consistently demonstrated low-level (0 or 1+) expression (Figure 3). Typical examples of the staining pattern observed in cases in the ALCL and other TCL categories are shown in Figure 2.

Statistical analysis using the Kruskal–Wallis test with Dunn’s multiple comparison confirmed that mean PRAME scores were significantly higher in CHL compared to ALK-negative ALCL, all ALCL, and other TCL cases (*p* < 0.01). Furthermore, PRAME expression proved to be a highly specific marker for CHL (Table 2). A 3+ score demonstrated a specificity of 98% (95% CI: 87–100%) and a positive predictive value (PPV) of 95% (95% CI: 73–99%) for identifying CHL. While the sensitivity increased to 92% (95% CI: 73–99%) when using a threshold of at least 1+ staining, the specificity dropped significantly to 68% (95% CI: 52–82%), indicating that higher-intensity (at least 2+) staining is the most reliable diagnostic parameter. Fisher’s exact test results for all scoring thresholds (3+, at least 2+, and at least 1+) remained statistically significant at *p* < 0.01 (Table 2).

**Table 2:** Performance of PRAME protein expression for identifying classic Hodgkin lymphoma.

PRAME Expression	Sensitivity	Specificity	PPV	NPV	Fisher’s Exact Test (*p*)
3+ (%, 95% CI)	79 (58–93)	98 (87–100)	95 (73–99)	90 (79–95)	<0.01
At least 2+ (%, 95% CI)	88 (68–97)	90 (77–97)	83 (67–93)	93 (81–97)	<0.01
At least 1+ (%, 95% CI)	92 (73–99)	68 (52–82)	63 (52–73)	93 (79–98)	<0.01

## 4. Discussion

Our study demonstrates that PRAME is frequently expressed in CHL HRS cells. Given its restricted expression in normal tissues and its relatively clean nuclear staining pattern, our results join the growing body of literature positioning PRAME as a significant marker for diagnosis [18].

This study has several limitations. First, its retrospective nature meant relying on existing medical records, which may have led to incomplete data or potential selection bias. The inclusion of only a few cases in the category of “other T-cell lymphoma” limits the statistical power and the ability to draw definitive conclusions for non-ALCL T-cell lymphomas. Future investigation could expand the cohort of confirmed AITL cases, as this type of lymphoma is a critical morphologic mimic of CHL. Additionally, this study does not directly compare, by methods such as head-to-head or multivariable assessment, the existing diagnostic stain panel with a panel including PRAME. This study did not directly assess the degree of diagnostic improvement of adding PRAME to morphology, PAX5, CD15, CD20, CD45, CD43, EBER ISH, and T-cell markers. The absence of PRAME staining or a low level of such is not diagnostic of a T-cell neoplasm. The exclusion of core biopsies for CHL in this study limits extrapolation of the findings for real diagnostic use in the setting of core biopsies of CHL where the number of HRS cells may be limited. Finally, the limited number of other confirmed subtypes of CHL besides nodular sclerosis may limit the extrapolation of this study’s findings to non-nodular sclerosis cases.

The most critical limitation of this study is that we did not seek to identify the exact lineage of the PRAME-positive cells in each case. As archival case slides varied in terms of whether a full immunohistochemical panel of B- and T-cell markers was performed, we did not seek to match the PRAME-positive cells in our study with definitive B- or T-cell staining. This information would be especially informative for the ALCL and TCL in the study, as it would reveal whether the PRAME staining that was observed corresponded to neoplastic T cells or to intermixed B cells such as B-immunoblasts in nodal T-follicular helper cell lymphomas. This study also does not correlate EBV staining patterns with PRAME results.

**Figure 4:**
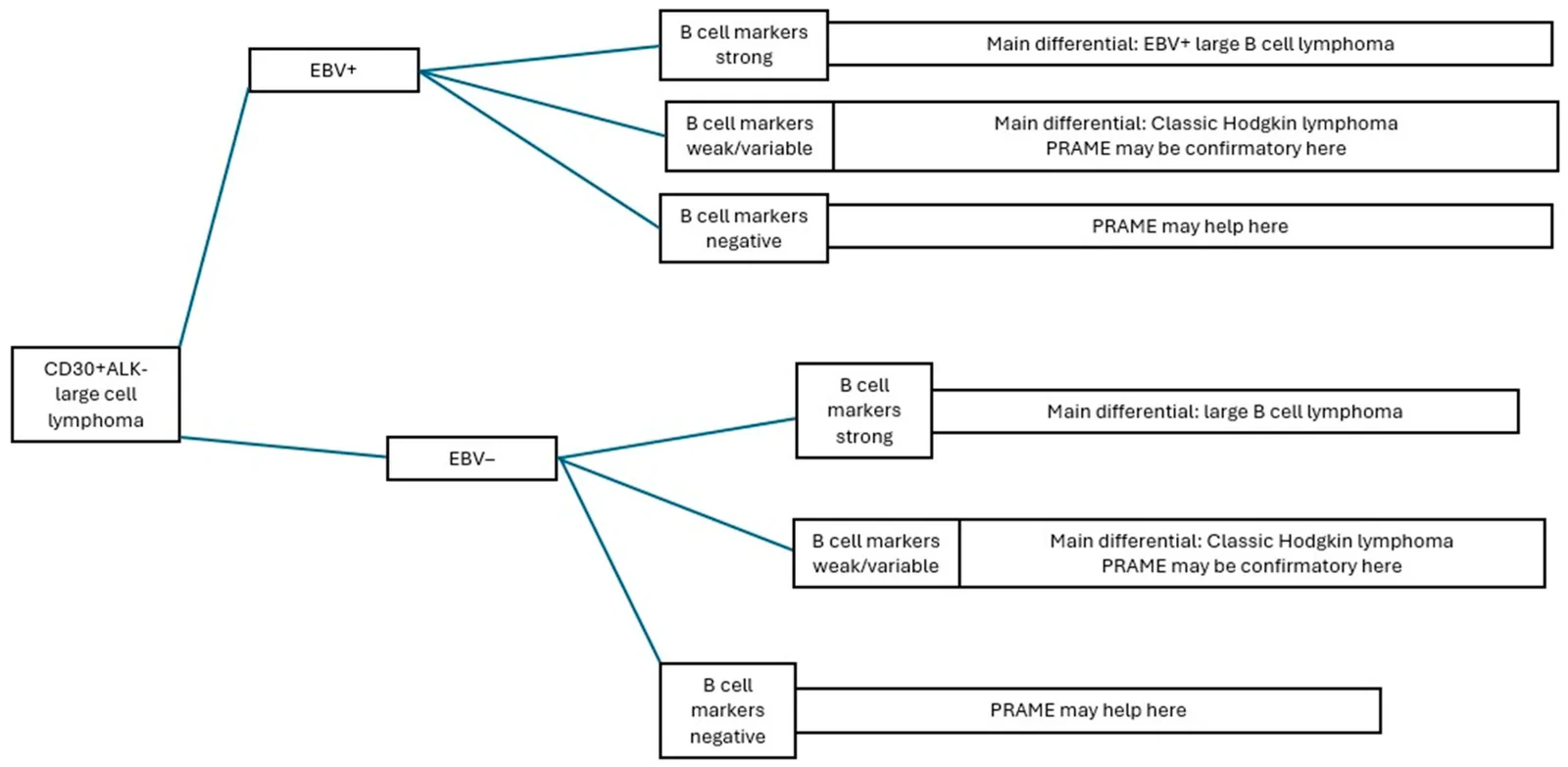
Proposed diagnostic algorithm for use of PRAME.

The impact of our study is the identification of a potential additional marker to differentiate CHL from its morphologic T-cell mimics. Commonly used Hodgkin panel markers that would support a CHL diagnosis, if positive, are CD30, PAX5, MUM1, CD15, and EBER in situ hybridization (EBER ISH). The absence of CD20, CD45 and CD43 are also supportive diagnostic features. CD30 and MUM1 expression can be found in the large cells of the morphologic mimics and are not specific to CHL. CD15 and EBER ISH positivity are neither required for nor specific to CHL [19]. Weak PAX5 expression and a lack of expression of CD20, CD45 and CD43 therefore become crucial stains upon which the diagnosis relies. PAX5 can be impacted by technical artifacts including laboratory titration levels impacting the intensity of staining. PAX5 negativity can also be seen in a minority of CHL [19]. CD43 and CD45 expression can be difficult to assess in large cells due to a high number of background T cells normally expressing these two markers. CD20 can be expressed by a proportion of CHL cells [19], and the assessment of its expression can suffer from sampling error within small biopsies. An additional confirmatory marker expected to be positive in most CHL would give the pathologist another tool in this diagnostic setting. A proposed diagnostic algorithm is shown in Figure 4. PRAME would be most useful in the setting of a large cell lymphoma that is CD30-positive and ALK-negative. PRAME could potentially be used as a confirmatory test for CHL diagnoses, in the setting of unusual staining patterns that are not typical of a particular lymphoma diagnosis, or in a scenario where ALCL or other TCL is a significant diagnostic consideration.

One of the scenarios that requires high diagnostic urgency is the syncytial variant of nodular sclerosis CHL. This variant is characterized by cohesive sheets of HRS cells and geographic necrosis, which can mimic metastatic carcinoma or melanoma [20]. Because the syncytial variant often presents with a more advanced clinical stage and bulky disease, a fast and accurate diagnosis is essential to avoid the delays associated with misidentifying this aggressive morphology as a non-lymphoid malignancy [21,22]. However, the confluent growth pattern can obscure the typical inflammatory background, complicating the initial biopsy interpretation [22,23]. Due to the long TAT for molecular tests, identifying reliable IHC markers like PRAME may help bridge this gap while molecular results are pending.

Furthermore, with regard to the T-cell molecular testing that is sometimes required to resolve the CHL-versus-T-cell-lymphoma question, access to molecular testing is not universal; many community and regional laboratories lack the necessary equipment and expertise, forcing specimen send-outs that further prolong the diagnostic timeline [24]. These limitations highlight the need for more immunophenotypic markers that have shorter turnaround times.

PRAME belongs to a group of CTAs that are predominately expressed in the normal testis and a variety of tumors, and are involved in immunity and reproduction [25,26]. Like other CTAs, PRAME is abundant in a wide variety of malignant tumors [27]. It has been reported that PRAME appears to serve as an inhibitor of retinoic acid receptor (RAR) signaling via SOX9 and upregulation of PRAME contributes to tumorigenesis by inhibiting the RA/RAR signaling pathway [10,13]. The decreased expression of PRAME inhibits cell growth and induces cell apoptosis by activating the p53/B-cell lymphoma 2 (Bcl-2)-mediated apoptosis pathway and increasing p21 expression [28].

Some ALK-negative ALCLs harbor *DUSP22* or *TP63* rearrangements [29]. Although our study does not explore the specific relationship between PRAME and these molecular lesions, this could be further investigated. Interestingly, the loss of PRAME has been previously noted in multiple B-cell malignancies, especially chronic lymphocytic leukemia [30,31,32]. In contrast, our study shows that Hodgkin cells preferentially express PRAME.

Looking beyond diagnosis to therapy, the recent literature identifies PRAME as a promising target for novel interventions. PRAME overexpression has been documented in a significant proportion of classic Hodgkin lymphomas and diffuse large B-cell lymphomas (DLBCLs) [12,33,34], where it correlates with tumor microenvironments and clinical outcomes [12,15,16,35]. Its restricted expression in normal tissues makes it an attractive candidate for immunotherapies, including PRAME-specific TCR-T-cell therapies [36], which are currently being explored for refractory hematologic malignancies [37].

## 5. Conclusions

In conclusion, while PRAME is not lineage-specific and should be interpreted in the context of morphology, a full immunohistochemical stain panel, EBV studies and molecular testing, when indicated, our study shows that PRAME immunohistochemistry is a potential additional diagnostic tool for distinguishing classic Hodgkin lymphoma (CHL) from anaplastic large cell lymphoma (ALCL) and other T-cell lymphomas. We found that strong, diffuse nuclear PRAME expression (at least 2+) is characteristic of the majority of CHL cases, whereas it remains largely absent in ALCL and other T-cell lymphomas in our study. PRAME may serve as a valuable adjunct to existing Hodgkin workup stain panels in the diagnostic timeframe before confirmatory molecular TCR testing is available.

## Figures and Tables

**Table 1:** Characteristics of patient demographics.

	CHL	ALCL (ALK−)	ALCL (ALK+)	Other T-Cell Iymphoma
Cases (N)	24	21	9	11
Male (N)	9	12	6	8
Female (N)	15	9	3	3
Age (mean ± SD, years)	37.1 ± 23.4	60.7 ± 9.4	24.4 ± 15.9	56.8 ± 7.9

## Data Availability

The raw data supporting the conclusions of this article will be made available by the authors on request.
